# False Alarm Reduction in Self-Care by Personalized Automatic Detection of ECG Electrode Cable Interchanges

**DOI:** 10.1155/2020/9175673

**Published:** 2020-01-27

**Authors:** Jocelyne Fayn, Paul Rubel

**Affiliations:** SFR Santé Lyon-Est: eTechSanté, INSERM US7, Université de Lyon, Lyon 69372, France

## Abstract

**Introduction:**

False alarm reduction is an important challenge in self-care, whereas one of the most important false alarm causes in the cardiology domain is electrodes misplacements in ECG recordings, the main investigations to perform for early and pervasive detection of cardiovascular diseases. In this context, we present and assess a new method for electrode reversals identification for Mason-Likar based 3D ECG recording systems which are especially convenient to use in self-care and allow to achieve, as previously reported, high computerized ischemia detection accuracy.

**Methods:**

We mathematically simulate the effect of the six pairwise reversals of the LA, RA, LL, and C2 electrodes on the three ECG leads I, II, and V2. Our approach then consists in performing serial comparisons of the newly recorded 3D ECG and of the six derived ECGs simulating an electrode reversal with a standard, 12-lead reference ECG by means of the CAVIAR software. We further use a scoring method to compare these analysis results and then apply a decision tree model to extract the most relevant measurements in a learning set of 121 patients recorded in ICU.

**Results:**

The comparison of the seven sets of serial analysis results from the learning set resulted in the determination of a composite criteria involving four measurements of spatial orientation changes of QRS and T and providing a reversal identification accuracy of 100%. Almost the same results, with 99.99% of sensitivity and 100% of specificity, were obtained in two test sets from 90 patients, composed of 2098 and 2036 representative ECG beats respectively recorded during PTCA balloon inflation, a procedure which mimics ischemia, and before PTCA for control.

**Conclusion:**

Personalized automatic detection of ECG electrode cable interchanges can reach almost the maximal accuracy of 100% in self-care, and can be performed in almost real time.

## 1. Introduction

According to the World Health Organization, ischemic heart disease is still the leading cause of death in the world [1], and the electrocardiogram, which can be easily recorded anywhere, at any time, and at low cost, remains the main source of information for the early detection of myocardial ischemia or infarction [2]. Prehospital ECGs are highly recommended to reduce the time to diagnosis and treatment, and it is further advised to perform serial computer-assisted ECG recordings to improve the detection of dynamic ECG changes [2]. With the development of pHealth and mHealth strategies, various smart ECG devices solutions have been proposed for the pervasive detection of cardiac events from the first symptoms. The personal self-care system we designed and developed at the beginning of the 2000's [3], allows any citizen to record high quality ECGs on demand with a portable device, called PEM for personal ECG monitor, which is endowed with self-adaptive embedded intelligence, mobile health record management support on smart-media card, embedded web server, and wireless communication. The EPI-MEDICS solution, which stands for Enhanced Personal, Intelligent, and Mobile system for Early Detection and Interpretation of Cardiac Syndromes, provides ambient intelligent and pervasive computing services for a reliable detection of life-threatening myocardial ischemia and arrhythmia, and alarms management [4]. Only four active electrodes, that are easy to set-up on the thorax by the patients themselves, are needed to retrieve the spatiotemporal information of the electrical activity of the heart, which is necessary and sufficient for accurate ischemia detection [5, 6]. These electrodes are LA (left arm), RA (right arm), and LL (left leg) or LF (left foot), also called as L, R and F, which are placed in the Mason-Likar position, and the precordial C2, yielding for a pseudo-orthogonal 3D ECG (I, II, V2). We demonstrated that a diagnostic accuracy of 98%, with 98% of sensitivity and specificity, can be obtained for ischemia detection by using these three standard ECG leads, thus exceeding by 23% the performance of the standard ECG criteria [6].

The main problem to solve at present in self-care is to reduce the number of false alarms which may be caused by ECG electrode cables reversals. The incidence of the electrode cables misplacements on the diagnosis accuracy and the importance of their automatic detection have been largely reported, even for ECGs recorded at the hospital [7–11]. All these authors highlight the increasing number of medical errors which are responsible for deaths, in particular in the US, ECG cable reversal being one prevalent reason. Incorrect electrode configurations can simulate rhythm or conduction disturbances, myocardial ischemia or infarction [11]. Bond et al. demonstrated that the clinical diagnosis is affected in 17–24% of patients, and STEMI diagnosis is missed in 11% [9]. Nilsson et al. found that the incidence of reversals for ECGs in the emergency department was twice that of those obtained within the hospital [10]. They have also shown that the most common reversal is of the right and left arm electrode cables. Various methods of detection have been published. Hedén et al. developed an artificial neural network for the recognition of right/left arm lead reversal, which is known to be the easiest to detect. They obtain a high specificity of about 99.9%, and a sensitivity of 98.7% [12]. Kors and van Herpen obtained similar results for the RA-LA and RA-LL reversals by using the correlation between recorded and reconstructed ECG leads, but only a sensitivity of 17.9% for the LA-LL interchange [13]. Han et al. have shown that the same algorithm based on a decision tree classifier may be used for conventional and Mason-Likar electrode placements [14]. They obtained a sensitivity of 91.3% and 72.8% for respectively the LA-RA and RA-LL reversals with a specificity of 97.9% and 97.5% for conventional ECGs, versus 88.9% and 75.9% of sensitivity with 96.5% and 98.5% of specificity for Mason-Likar ECGs. Using linear support vector machine, these authors found a sensitivity and a specificity of 56.5% and 99.9% for detecting seven precordial cable interchanges (V1-V2, V1–V3, V2-V3, V3-V4, V4-V5, V4–V6, and V5-V6), and respectively 93.8% and 99.9% for limb cable interchanges, but they did not consider the LA-LL interchange which requires serial ECGs in their opinion [15]. Jekova et al. considered in their study, based on interlead correlation, four LA, RA and LL reversals (RA-LA, LL-RA, clockwise and counter-clockwise), and the 15 pairwise precordial leads [16]. Their results in three test sets vary from 91.7% to 97.6% of sensitivity and from 99.2% to 99.7% of specificity for RA-LA and LL-RA interchanges. We found no studies involving reversals between the limb leads and the precordial leads.

The aim of this paper is to present a new method able to automatically detect a possible electrodes reversal during an ECG recording, intended, in case of the occurrence of changes of the recorded ECG in comparison with a previously recorded reference ECG which is stored in the personal device, to recommend in real time to the user of the device to check if there is any electrode cable interchange for the detected electrode pair(s). The concept of the EPI-MEDICS solution is indeed to store in the ECG device both a standard 12-lead ECG as well as a 3D reference ECG, recorded at some minutes or hours of interval, to which any new ECG will be compared for the detection of serial ECG changes, as recommended by the international societies in cardiology. The reference ECGs should be validated by a physician.

In this paper, we mathematically simulate all the six possible pairwise electrode reversals on the 3D ECGs, including the interchanges between limb and precordial leads, with the challenge of finding, in a learning set, powerful detection criteria for self-care situations, which are then assessed in two independent test sets.

## 2. Materials and Methods

### 2.1. Study Populations

Three data sets were considered. The learning set is composed of 170 pairs of rest ECGs of 10 s duration, a standard 12-lead ECG and a 3D ECG, recorded on 121 patients admitted in the ICU of the Lyon Cardiovascular Hospital, France. All pairs were recorded the same day, except for one pair which was recorded at 6 days interval. The acquisition time difference of the 169 pairs of ECGs is in average 40 min 28 s with a standard deviation of 30 min 50 s (Min: 2 min 9 s, Max: 1 hour 16 min 29 s). 97 pairs of ECGs have been recorded by one technician and the other 73 pairs by another technician. The 121 patients are composed of 83 men and 38 women, aged of 61 years in average with a standard deviation of 15 years (Min: 18, Max: 87), and were admitted for coronary heart diseases and/or arrhythmias. The 12-lead ECGs were acquired with a legacy electrocardiograph, and the 3D ECGs with our PEM device, both at a sampling rate of 500 samples/s with an amplitude resolution of 5 *µ*V. Written informed consent was obtained from each patient.

The two test sets respectively called C and I are continuous 12-lead ECG recordings performed in 90 coronary patients who underwent elective percutaneous transluminal coronary angioplasty (PTCA) at the Charleston Area Medical Center, WV. The ECGs were recorded during balloon inflation that mimics myocardial ischemia (test set I), and before PTCA in the catheterization laboratory for control (test set C), within the framework of the STAFF Studies Investigations approved by the Investigational Review Board. Informed consent was obtained from each subject. We already described this database [5, 6] which includes four locations of balloon inflation: left anterior descending coronary artery (LAD) for 31 patients, left circumflex coronary artery (LCX) for 15 patients, left main coronary artery (LM) for 2 patients and right coronary artery (RCA) for 42 patients. The duration of the control recordings was 5–10 min, and the mean duration of the balloon inflation was 4 min 28 s (SD: 1 min 15 s), resulting in more than 2000, 10 s duration ECG recordings per test set.

### 2.2. Simulation of Electrode Cables Reversals

**Figure 1:**
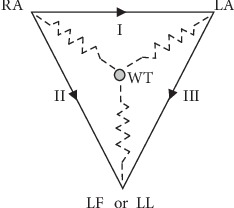
The Einthoven triangle with the schematic representation of the Wilson central terminal.

We mathematically simulated all six possible pairwise electrodes reversals of the four considered electrodes: LA, RA, LL, and C2 (for V2) by using the standard equations defining the ECG leads, according to the Einthoven triangle (Figure 1). Let us call I, II, III, and V2 the original recorded leads, and I^∗^, II^∗^, III^∗^, and V2^∗^ the modified ECG leads, after having applied the specified electrode cables pair reversal. Let us also denote VL, VR, VF, VC, and VW the unipolar potentials of the exploring electrodes LA, RA, LL, C2, and of the Wilson central terminal WT for writing simplification. These potentials are defined as follows:

(1) $$\begin{array}{c} \text{VL}=\text{VW}+\left(\frac{2\text{I}-\text{II}}{3}\right), \end{array}$$

(2) $$\begin{array}{c} \text{VR}=\text{VW}-\left(\frac{\text{I}+\text{II}}{3}\right), \end{array}$$

(3) $$\begin{array}{c} \text{VF}=\text{VW}+\left(\frac{2\text{II}-\text{I}}{3}\right), \end{array}$$

(4) $$\begin{array}{c} \text{VC}=\text{VW}+\text{V}2, \end{array}$$

(5) $$\begin{array}{c} \text{VW}=\frac{\text{VL}+\text{VR}+\text{VF}}{3}. \end{array}$$

#### 2.2.1. LA and RA Electrodes Reversal

(6) $$\begin{array}{c} {\text{I}}^{*}=-\text{I,} \end{array}$$

(7) $$\begin{array}{c} {\text{II}}^{*}=\text{III}=\text{II}-\text{I}. \end{array}$$

#### 2.2.2. LA and LL Electrodes Reversal

(8) $$\begin{array}{c} {\text{I}}^{*}=\text{II,} \end{array}$$

(9) $$\begin{array}{c} {\text{II}}^{*}=\text{I}. \end{array}$$

#### 2.2.3. RA and LL Electrodes Reversal

(10) $$\begin{array}{c} {\text{I}}^{*}=-\text{III}=\text{I}-\text{II}, \end{array}$$

(11) $$\begin{array}{c} {\text{II}}^{*}=-\text{II}. \end{array}$$

#### 2.2.4. LA and C2 Electrodes Reversal

(12) $$\begin{array}{c} {\text{I}}^{*}=\text{VC}-\text{VR}=\text{VW}+\text{V}2-\text{VR}=\text{V}2+\left(\frac{\text{I}+\text{II}}{3}\right), \end{array}$$

(13) $$\begin{array}{c} {\text{II}}^{*}=\text{II,} \end{array}$$

(14) $$\begin{array}{c} {\text{VW}}^{*}=\frac{\text{VC}+\text{VR}+\text{VF}}{3}, \end{array}$$

(15) $$\begin{array}{c} \text{V}2^{*}=\text{VL}-{\text{VW}}^{*}, \end{array}$$

(16) $$\begin{array}{c} \text{Thus: V}2^{*}=\text{VW}+\left(\frac{2\text{I}-\text{II}}{3}\right)-\left(\frac{\text{VC}+\text{VR}+\text{VF}}{3}\right)=-\left(\frac{\text{V}2}{3}\right)+4\left(\frac{2\text{I}-\text{II}}{9}\right). \end{array}$$

#### 2.2.5. RA and C2 Electrodes Reversal

(17) $$\begin{array}{c} {\text{I}}^{*}=\text{VL}-\text{VC}=\left(\frac{2\text{I}-\text{II}}{3}\right)-\text{V}2, \end{array}$$

(18) $$\begin{array}{c} {\text{II}}^{*}=\text{VF}-\text{VC}=\left(\frac{2\text{II}-\text{I}}{3}\right)-\text{V}2, \end{array}$$

(19) $$\begin{array}{c} {\text{VW}}^{*}=\frac{\text{VC}+\text{VL}+\text{VF}}{3}, \end{array}$$

(20) $$\begin{array}{c} \text{Thus}:\text{V}2^{*}=\text{VR}-{\text{VW}}^{*}=\text{VW}-\left(\frac{\text{I}+\text{II}}{3}\right)-\left(\frac{\text{VC}+\text{VL}+\text{VF}}{3}\right). \end{array}$$

By replacing VC by (VW + V2) and ((VL + VF)/(3)) by (VW − (VR/3)), we obtain:

(21) $$\begin{array}{c} \text{V}2^{*}=\left(\frac{-\text{V}2}{3}\right)-4\left(\frac{\text{I}+\text{II}}{9}\right). \end{array}$$

#### 2.2.6. LL and C2 Electrodes Reversal

(22) $$\begin{array}{c} {\text{I}}^{*}=\text{I,} \end{array}$$

(23) $$\begin{array}{c} {\text{II}}^{*}=\text{VC}-\text{VR}=\text{V}2+\left(\frac{\text{I}+\text{II}}{3}\right), \end{array}$$

(24) $$\begin{array}{c} {\text{VW}}^{*}=\frac{\text{VC}+\text{VR}+\text{VL}}{3}, \end{array}$$

(25) $$\begin{array}{c} \text{Thus}:\text{V}2^{*}=\text{VF}-{\text{VW}}^{*}=\left(\frac{-\text{V}2}{3}\right)+4\frac{\left(2\text{VF}-\text{VL}-\text{VR}\right)}{9}=-\left(\frac{\text{V}2}{3}\right)+4\frac{\left(-\text{I}+2\text{II}\right)}{9}. \end{array}$$

### 2.3. ECG Analysis

Our reasoning is the following: each time a 3D ECG is recorded in self-care, all potential reversal simulations shall be performed in almost real time. If the 3D ECG has been correctly recorded, the serial ECG changes between the simulated 3D ECGs and the standard reference ECG, called Δ*s*, should be greater than the ECG changes between the recorded 3D ECG and the standard reference ECG, called Δ*r*. We thus perform a serial ECG analysis between the standard 12-lead reference ECG of each patient and seven 3D ECGs, the recorded one and the 3D ECGs which are derived by applying the mathematical transformations defined in Section 2.2 and respectively simulating the six pairwise electrode cables reversals, for each of the three datasets.

For the learning set, 7 × 170 ECG comparisons were performed. The standard 12-lead ECGs were pre-processed with median beat computation and ECG waves delineation by means of the manufacturer specific software of the ECG acquisition system, and the 3D ECGs were preprocessed and a typical beat was delineated by the Lyon program [17] which is embedded in the PEM devices. The two acquiring devices being compliant with the SCP-ECG standard communication protocol, ECG signal data, metadata and analysis results viz beat typification, wave delineation and global measurements data were retrieved as SCP-ECG files [18, 19]. Serial analyses have thus been performed between the reference median beats of the standard 12-lead ECGs and the typical beats of the 3D ECGs.

Concerning the test sets C and I, the continuous control and during inflation recordings were split into successive, 10 s duration ECG sequences which were then preprocessed and analyzed by the Lyon program, and encoded in the SCP-ECG format. A serial beat-to-beat analysis was next performed for each patient between a typical beat from the control recording that was selected as the reference beat, and all the other typical beats from the control recording and from the recording which has been acquired during balloon inflation. Thus, a total of 2036 serial beat-to-beat comparisons were performed on test set C (control recordings) and 2098 on test set I (recordings performed during inflation and showing ischemia changes induced by angioplasty) for the 90 patients. This serial processing was again repeated for the six reversal simulations.

Serial ECG analysis was performed with our CAVIAR program [5, 6] which is now available on-line at the following address: http://fr-ecg.univ-lyon1.fr/ [20]. The CAVIAR software performs an optimal spatiotemporal comparison of two median or typical ECG beats. For this study, we only analyzed the three leads I, II, and V2 for all the ECGs of our study populations in order to compare the same leads of the standard ECGs and of the 3D ECGs. For serial ECG processing, we are using –V2/2 as third axis in order to get an almost orthonormal representation system of the electrical activity of the heart.

### 2.4. Electrode Cables Reversal Detection Criteria Determination

The determination of the criteria for electrode cables reversal detection was based on the analysis of a series of CAVIAR serial measurements and of the differences between the values Δ*s* obtained when comparing the 3D ECGs simulating a given reversal with the reference ECGs and the values Δ*r* found with the recorded 3D ECGs, in the learning set. A ranking score of classification of the observations “ECGs with reversal” versus “recorded ECGs” was computed. A dichotomization decision tree model was then applied to extract the most performant measurements.

### 2.5. Statistical Analysis

Sensitivity, representing the percentage of correct detection of electrode cables reversals, is computed in test set I by comparing the values Δ*r* with the values Δ*s* for each of the six interchange simulations. Indeed, the study of sensitivity supposes that the considered ECGs are not correctly recorded, which we simulate by applying the six different reversal transformations. Then, by reapplying the same lead transformations on these ECGs, we get the original, recorded ECGs which are supposed to be correctly acquired in our study material as they have been checked by a cardiologist at the time of recording. The cable reversal functions we describe in this paper are mathematically defined as involutions. Specificity is figured out in test set C by comparing the values Δ*s* with the values Δ*r* and then by computing the percentage of serial comparisons for which the reversal detection criteria classifies the original, recorded ECGs as correct.

## 3. Results

Four CAVIAR measurements occurred to be the most relevant for any pairwise electrode reversals detection in the learning set. These serial measurements between the standard reference ECG and the 3D ECG are the orientation changes of the QRS (OCQ) and repolarization (OCR) waves, the orientation changes of the initial part of QRS (OCI) and the angular deviation of the preferential plane of the terminal part of QRS (TPPAD). The orientation changes (OC) are measured by the spatial angular deviation of the center of gravity of the considered wave or part of the wave. The initial and terminal parts of QRS correspond to about the first and last 30 ms of the QRS wave. Their determination was previously reported in [21, 22]. The following criteria:

*If, for a given electrode pair reversal simulation, Δs > Δr for any of the OCQ, OCI, OCR or TPPAD measurements, then the hypothesis of the reversal of the given electrode cables pair in the acquired 3D ECG is rejected*

Results in 100% of sensitivity and in 100% of specificity for the 6 × 170 = 1020 serial comparisons that have been performed, patient by patient, in the learning set.

In the test phase, almost the same 100% of total accuracy results are obtained when applying the previously defined criteria. Specificity is 100% for the 12 216 (=2036 × 6) beat-to-beat comparisons results of the simulated reversals for the control recordings group (C). Sensitivity is 99.99% for the 12 588 (=2098 × 6) simulated reversals comparison results obtained in the inflation group (I). Only one LA-LL electrodes reversal was not detected. Sensitivity was 100% for the other five pairwise electrode cables interchanges.

Table 1 summarizes the relative contribution of each selected measurement for each of the six electrodes reversals simulations. These results show that OCQ and OCI are the most relevant for all reversals, and that TPPAD mainly contributes to the detection of the LA and LL electrode cables interchange.

## 4. Discussion

Our study anew demonstrates the relevance of personalized medicine empowered by patient-specific data analysis algorithms aiming at enhancing the diagnostic accuracy and the detection of technical errors. All six possible pairwise electrode cables reversals were detected with our approach with an accuracy of almost 100%, thus providing the yet best reported results. Only four measurements related to the QRS and T waves were involved. They can be computed in less than 0.04 s per serial comparison. As an example, the total processing time for the comparison of the 2098 typical beats from dataset I with the respective control reference beats is 77 s with a standard 3 GHz PC with 4 GB memory, and it is 25 s for the 170 × 4 = 680 comparisons of the 170 original 3D ECGs from the learning set and of the 170 × 3 3D ECGs simulating the studied C2 electrode cable misplacements. Such an electrode cable interchange detection can therefore be performed in almost real time, allowing a significant reduction of false alarms. In addition, the presented method allows, in case of a reversal detection, to indicate to a self-care ECG device user which electrode cables misplacements need to be checked.

**Figure 2:**
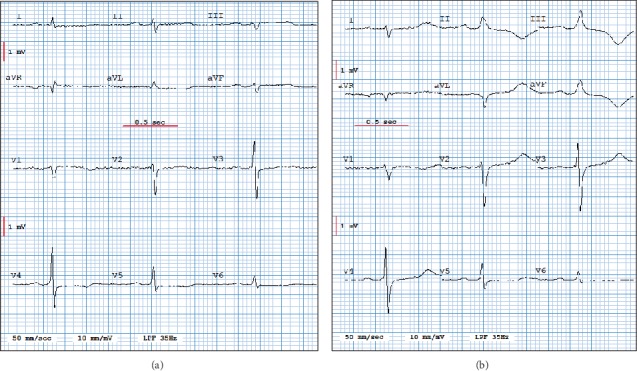
Filtered ECG tracings of a patient undergoing an angioplasty, respectively recorded at 5 min 0 s (Panel (a)) and 5 min 20 s (Panel (b)) after balloon inflation. These tracings clearly show QRS pattern changes in leads I and II in panel (b) that may be interpreted as a reversal of these leads with respect to Panel (a) and explain why the ECG displayed in Panel (b) was misclassified by our LA–LL electrode cables reversal detection method.

The only one 10 s ECG for which the simulated LA–LL electrode cables reversal was not detected by our method is a dataset I ECG sequence recorded 5 min 20 s after balloon inflation in the patient's proximal right coronary artery. All other simulated LA–LL reversals of all the previous and following 10 s ECG sequences from this dataset I patient were detected. The incorrectly classified ECG sequence was extremely noisy and the visual aspect of the QRS pattern highlights changes that mimic a LA–LL electrode cables reversal, as shown in Figure 2. However, such an error is not detrimental in the case there are ischemic changes that will be detected anyway.

We are convinced that the performance we obtain in this study in comparison with the previously reported results on the same topic is mainly the result of using serial ECG analysis, which is besides highly recommended by the international societies of cardiology, even for expert cardiologists. Our method still needs to be assessed on other large databases in order to evaluate its robustness and reliability. But, unfortunately, annotated and well documented serial ECG databases are not yet available, although only standard 12-lead ECGs or 3-lead ECGs (I, II, V2) are necessary.

Concerning the LA–LL reversal, which is usually reported as the most difficult to detect [13, 15], we can see in Table 1 the importance of the TPPAD angular deviation of the principal plane of the terminal part of QRS, for its detection. This parameter gives the highest percentage of correct classifications for this reversal when we compare a Mason-Likar ECG with a standard ECG in the learning set and, during balloon inflation, when we compare an ECG which is altered by changes similar to ischemia effects with a reference ECG.

In addition, the orientation changes of the repolarization phase (OCR) mostly contribute to the identification of reversals between the precordial electrode and the peripheral electrodes, which is an interesting result because we found no studies considering this kind of reversal for which we provide in this paper unpublished new simulation formulas. Spatial orientation changes of the beginning of QRS (OCI) seem to be the most powerful measurement but nevertheless the global orientation changes of the whole QRS loop (OCQ) are complementary.

**Figure 3:**
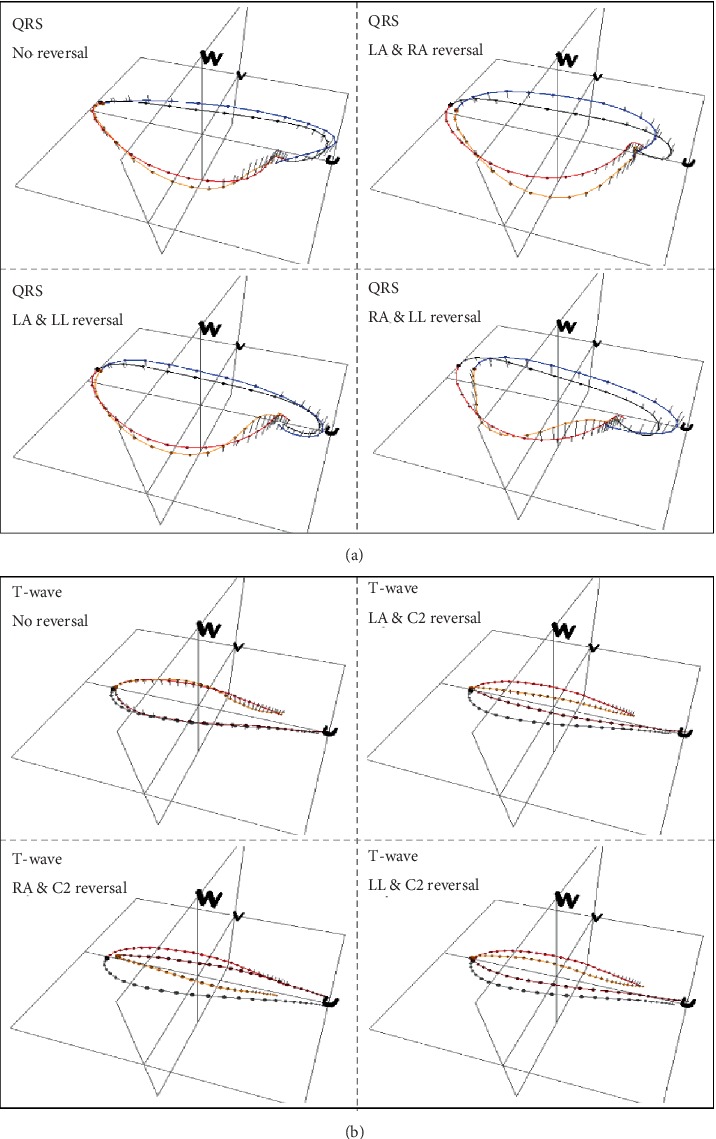
Sample CAVIAR displays of serial QRS (a) and T wave (b) changes for the tested electrode cable reversals with reference to the standard ECGs of patients (a) and (b) presented in Figure 4. The first display at the top left of panels (a) and (b) shows the CAVIAR optimal superimposition results between the standard ECGs and the corresponding, electrode cable reversals free, original 3D ECGs recorded with the PEM device at respectively 5 min (patient (a)) and 1 h interval (patient (b)). The three other displays of panel (a) highlight the effect on the QRS wave of a simulated, pairwise LA, RA, and LL electrode cables reversal on the PEM ECGs of patient (a), and in panel (b) the effect on the T wave of a reversal between each of the limb electrodes and C2 for patient (b). The beginning and the end of the QRS wave (resp. of the T wave) are respectively colored in black and red (resp. in red and grey) for the standard reference ECG and in blue and orange (resp. in orange and dark red) for the PEM ECG.

**Figure 4:**
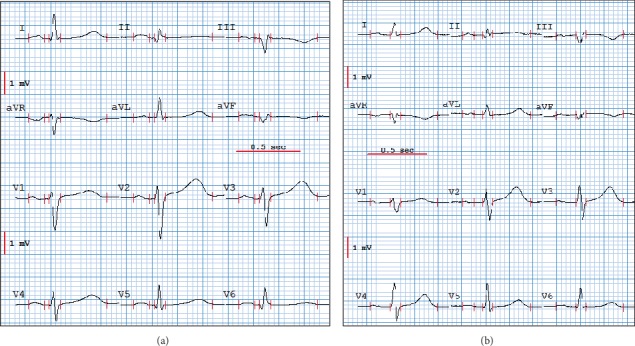
Standard 12-lead ICU ECG recordings of two patients respectively called (a) and (b) selected to illustrate our results of electrode cables reversals detection as displayed in Figure 3. Panel (a): 51-year-old man admitted for arrhythmia. Panel (b): 69–year-old man admitted for coronary disease.

Figure 3 displays an example of CAVIAR representations highlighting these types of ECG changes for two patients of the learning set recorded at their admission in the ICU (Figure 4) for arrhythmia (Patient (a)) and for coronary diseases (Patient (b)). We can see, Figure 3, that the spatial QRS and T loops of the standard 12-lead reference ECG and of the 3D ECG recorded with the PEM are very well superimposed in their preferential space (U, V, W) defined by the inertia axes of the spatial waves. We thus can visualize in 3D the spatiotemporal changes when we superimpose the recorded standard ECG and the simulated PEM ECG when an electrode cable reversal occurs. This figure demonstrates that the whole QRS loop is modified in case of a LA-RA reversal (OCQ varies from 7° to 98°) and of a RA-LL reversal (OCQ and OCI differences are 32° and 76°), and that mainly the end of QRS is changed for a LA-LL reversal (TPPAD goes from 9° to 68°). In addition to QRS changes, the repolarization wave is also significantly modified when there is a cable interchange between C2 and the limb leads. OCR changes between Δ*r* and Δ*s* are 46° (LA–C2), 129° (RA–C2), and 72° (LL–C2).

## 5. Conclusions

ECG electrode cables reversals, which are one of the main causes of false alarms in self-care, can be automatically detected in almost real time with a great reliability for 3-lead ECG recordings based on I, II, V2, a recording system that provides the whole necessary spatiotemporal information for ischemia and arrhythmia computerized diagnosis, and which is easy and convenient to use, especially when the limb electrodes are placed in the Mason-Likar positions. The new method presented in this paper, which is based on a personalized, comparative spatiotemporal ECG analysis with respect to a patient's reference ECG by means of the CAVIAR software, has reached 99.99% sensitivity and 100% specificity in the respective test sets. All six possible pairwise electrode cable interchanges of the four active electrodes could be identified by comparing the serial analysis results obtained for the original, last recorded ECG, and with the results obtained for this same ECG after having applied on each lead the mathematical transformations simulating the six different electrode cable reversals. Even the interchanges between the limb and the precordial electrodes and between the LA and LL electrodes, which are usually not studied because they are known to be difficult to assess, were considered in the present study. Only four spatial orientation changes measurements of the QRS and T waves and of the onset and of the end of the QRS wave need to be computed for the recorded and the six simulated ECGs, a task which can be achieved in less than 0.25 s. The computation is based on the determination of the center of gravity and of the preferential space of the spatial QRS and T loops and of the initial and terminal parts of QRS. In conclusion, using the proposed patient specific approach, a personalized automatic detection of electrode cables reversals can considerably reduce the number of ECG-based false alarms in self-care.

## Figures and Tables

**Table 1:** Percentage of correct classifications for each pairwise electrode cables reversal simulation and for each individual measurement in the learning set and in the two test sets, to be compared with the 100% or almost 100% accuracy obtained in the 3 datasets when combining the 4 measurements as described in the section results.

Measurement/electrode reversal	Learning set (*N* = 170)				Test set 1—control recordings (*N* = 2036)				Test set 2—during inflation (*N* = 2098)			
	OCQ	OCI	OCR	TPPAD	OCQ	OCI	OCR	TPPAD	OCQ	OCI	OCR	TPPAD
LA–RA	94.1	95.3	85.3	79.4	100.0	98.6	94.9	91.3	98.5	97.1	77.2	80.0
LA–LL	74.7	76.5	75.9	80.6	96.4	94.6	95.7	93.4	75.5	81.6	66.2	84.9
RA–LL	91.2	96.5	87.1	72.9	99.0	98.0	97.2	88.6	95.4	96.2	83.9	71.5
LA–C2	92.9	92.9	91.8	92.4	98.7	98.3	98.9	95.5	95.0	95.6	84.9	88.3
RA–C2	91.2	97.6	91.8	90.6	97.8	98.6	98.0	95.6	85.3	94.0	86.1	87.8
LL–C2	96.5	93.5	91.2	78.2	97.6	95.4	95.4	94.6	90.9	91.9	77.5	84.4

## Data Availability

The ECG recordings of the learning dataset used in this study are available from the authors upon request. These ECGs have been collected within the frame of the European EPI-MEDICS project [4]. The previously reported datasets C and I were used to test the findings of this study. These prior studies are cited within the text as references [5, 6]. These datasets are part of the STAFF III database which is now available at doi:10.13026/C20P4H or https://physionet.org/physiobank/database/staffiii/.
